# Effects of Foot Massage on Severity of Fatigue and Quality of Life in Hemodialysis Patients: A Randomized Controlled Trial

**DOI:** 10.30476/IJCBNM.2020.81662.0

**Published:** 2020-04

**Authors:** Hosein Habibzadeh, Osman Wosoi Dalavan, Leyla Alilu, Jon Wardle, Hamidreza Khalkhali, Aisan Nozad

**Affiliations:** 1 Department of Medical Surgical Nursing, School of Nursing and Midwifery, Urmia University of Medical Sciences, Urmia, Iran; 2 Department of Public Health, Australian Research Centre in Complementary and Integrative Medicine, School of Health, University of Technology Sydney, Sydney, Australia; 3 Department of Biostatistics and Epidemiology, Inpatient’s Safety Research Center, Urmia University of Medical Sciences, Urmia, Iran; 4 Department of Traditional Iranian Medicine, School of Medicine, Urmia University of medical Sciences, Urmia, Iran

**Keywords:** Almond oil, Chamomile, Fatigue, Hemodialysis, Massage

## Abstract

**Background:**

Despite the advances in treatment, fatigue is a common symptom experienced by many patients undergoing hemodialysis, and is associated with poor health-related quality of life. The aim of the present study was to explore the impact of foot massage with chamomile oil and almond oil on the severity of fatigue and quality of life of Hemodialysis patients.

**Methods:**

In these four parallel groups controlled clinical trial, 120 male patients under hemodialysis were randomly assigned to foot massage groups and control (30 in each group) from June 2016 to April 2017 in Urmia, Iran. foot massage using either chamomile oil, almond oil or no oils was provided to patients undergoing hemodialysis for two months. The primary outcome measures were the Fatigue Severity Scale (FSS) and secondary outcomes included quality of life using the Short-Form Quality of Life for Renal Patients questionnaire (KDQOL-SF). Data were analyzed using ANOVA, Tukey’s and paired t-test in SPSS the (Version 16) at the significance level P<0.05.

**Results:**

The mean FSS scores after the implementation of foot massage in all intervention groups were significantly lower than the control group (P=0.005). Mean KDQOL-SF scores after the intervention in all intervention groups increased compared to the control group, but this increase was not statistically significant (P=0.34).

**Conclusion:**

Foot massage appears to be effective in reducing fatigue and improving quality of life in patients undergoing hemodialysis. Further studies are needed to confirm and extend these results. Furthermore, involvement of patients’ companions as family caregivers in massage therapy can lead to continuation of this effective intervention at home.

**Trial Registration Number:** IRCT2016121731438N1.

## INTRODUCTION

The global prevalence of chronic renal failure (CRF) is significantly elevated. Reports indicate a 10% worldwide rate for this disease. ^1^ Hemodialysis is the most important treatment for chronic kidney disease as of the 3 million people undergoing replacement renal treatment, 2.5 million (80%) use hemodialysis. ^2^ Annually, the number of patients undergoing hemodialysis in Iran increases by about 16%. ^3^ They can experience a number of associated symptoms. Fatigue is a common symptom experienced by many patients undergoing hemodialysis, and is associated with poor health-related quality of life; it is also an important predictor for survival of hemodialysis patients. ^4^ A study in Iran showed that one-third (33.9%) of hemodialysis patients experience moderate fatigue, and 60.7% experience severe fatigue. ^5^ Fatigue affects the quality of life and self-confidence in patients undergoing hemodialysis, resulting in patients completing fewer activities and expending more energy for a minimum of daily activities. ^6^ Unfortunately, evaluation and management of fatigue, in comparison with other non-invasive and visible symptoms, are often neglected in clinical management of hemodialysis patients. ^7^ The concept of quality of life includes a person’s potential ability to actively participate in the activities of daily life, the ability to pursue their interests and their general sense of well-being. This concept is now considered a subjective multidimensional and dynamic concept. ^8^

Massage therapy is one of the traditional and complementary medical modalities used by hemodialysis patients. ^9^ Because stresses and psychological pressures can lead to or exacerbate a number of human ailments, and since there are 7,000 nerves in each leg, foot massage and stimulation of these neural cells is posited to relax the patient and reduce stress, and thus return the body to equilibrium. ^10^ According to the US National Center for Complementary and Integrative Health, nearly 18 million persons were massaged with herbal essential oils in 2009 alone, combining the therapeutic effects of plant medicines with massage. ^11^ The results of a systematic review in Iran showed that aromatherapy reduced some of the complications of hemodialysis, including anxiety, fatigue, pruritus, pain of arteriovenous fistula puncture, sleep quality, depression, stress, and headache. In one case, it improved the quality of life of hemodialysis patients. ^12^ Chamomile products have been considered by researchers because Chamomile extract contains 120 types of chemical compounds, which include chamomzolins, flavonoids and coumarins, with the most important active ingredients in it being chamomzolin, epiphanum and bisabavalol. Chamomile also appears to have anti-inflammatory and anti-oxidant activity. ^13^ Bitter almond contains essential fatty acids, such as oleic, linoleic, palmitic, and stearic acids14 that have no adverse effects on the end stage renal disease (ESRD) patients, particularly if it is applied topically. However, its therapeutic effects on fatigue and quality of life require further study. 

Therefore, the use of this natural substance, which has no adverse effects and is inexpensive, acceptable and accessible to patients, can be effective in reducing complications in hemodialysis patients. ^12^ In the literature review, no studies, however, were found that compare the effects of foot massage with chamomile oil and almond oil to reduce complications in hemodialysis patients. Therefore, given the complications and heavy costs of managing complications, the aim of the present study was to explore the impact of foot massage with chamomile oil and almond oil on the severity of fatigue and quality of life of Hemodialysis patients.

## MATERIALS AND METHODS

The current study is a randomized clinical trial with four parallel groups. Male patients undergoing hemodialysis were randomized to either a foot massage group (using either almond oil, chamomile oil or no oil) or control group. The trial was conducted from June 2016 to April 2017 and the participants were selected from dialysis patients referring to both educational and medical centers (Taleghani and Imam Khomeini) in Urmia, Iran(Hemodialysis centers are located in these two hospitals). To determine the sample size at 95% confidence level and 80% test power, with a mean difference of at least 1.3 and a standard deviation of 1.5 in the intervention group and 1.7 in the control group, based on the study of Cho et al. (2004) ^15^ and according to the sample size formula, we determined 25 patients in each group; considering the length of the study and the probability of attrition of the samples, the maximum sample size was 30 in each group and 120 in total. 

$n=\frac{{\left(Z_{1-\alpha /2}\right)}^{2}(S_{1}^{2}+S_{2}^{2})}{d^{2}}$

$n=\frac{7.95(1.5^{2}+1.7^{2})}{{\left(1.3\right)}^{2}}=24.17\approx 25$

Patients who met the inclusion criteria were invited to participate in the trial, and written consent was obtained. Inclusion criteria for participation in the trial were patients with CRF undergoing hemodialysis; willingness to participate in the study; after at least 6 months on hemodialysis; no presence of infectious diseases (including all types of hepatitis); no recent severe psychological problem (e.g. psychosis or mania); lack of attendance in similar training courses (including massage courses); age between 18 and 85 years; male gender (due to the male being the interventionist to eliminate potential intervention biases and considering the cultural issues of Iran); attendance in dialysis sessions at least 3 times per week; at least elementary school education; no history of sensitivity, arthritis, rheumatoid arthritis or joint and orthopedic problems; and lack of using sedative and analgesic and regenerative drugs. Exclusion criteria included unwillingness to continue participation in the study, kidney transplantation during the study, the onset of other illnesses, and withdrawal from the hemodialysis program. 

**Figure 1:**
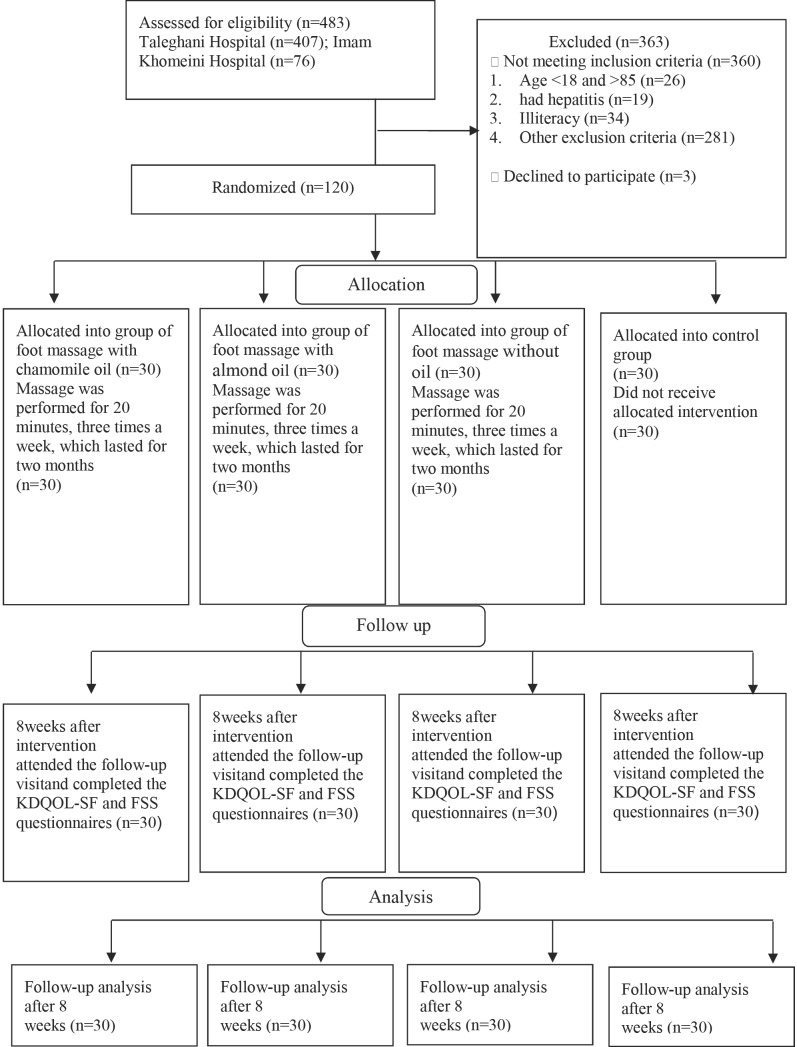
CONSORT flow diagram of the participants

Participants were randomly allocated into four groups (three intervention and one control group) by the first researcher. Numbers 1 through 120 were written on a small paper and placed in a basket; the participants were asked to take a number from the basket and classified based on this number (1 to 30 in the control group, 31 to 60 in the “Foot massage with chamomile oil group”, 61 to 90 in the “Foot massage with almond oil group” and 91 to the last in the “Foot massage without oil group”). This division was also randomly assigned (Figure 1).

Baseline characteristics were captured using a demographic questionnaire (age, marital status, educational level, economic status, type of insurance, duration of treatment with hemodialysis). To measure fatigue, we used the Fatigue Severity Scale (FSS) by Krupp (1989), ^16^ and quality of life was measured using the Kidney Disease Quality of Life-Short Form KDQOL-SF) by Hays (1997). ^17^ The FSS has 9 questions, and the range of scores for each question was from 1 to 7 (score 1 indicates that there is no fatigue, 2 to 4 moderate fatigue and higher than 4 indicates a severe fatigue). The total score range of the questionnaire was 9-63, so that a score of 36 or higher is an indication of fatigue. Hence, higher scores are indicative of higher fatigue. ^18^ In the present study, the mean fatigue score for each individual was considered as the final index, which is derived from the sum of the individual scores from different domains and divided by the number of questions, with values ranging from 1 to 7.

Based on Schwid et al.’s (2002) study, the FSS demonstrated good internal consistency, test-retest reliability, and responsiveness to treatment effects. Construct validity was supported by demonstration of associations between FSS scores and a visual analog scale rating of fatigue severity. Correlations were modest, however (r=0.47 in multiple sclerosis patients, r=0.50 in healthy adults). Thus, the FSS has limited face validity as a measure of fatigue severity. ^19^ Amtmann et al. (2012) obtained a Cronbach’s alpha coefficient of 0.93 for FSS. ^20^ In Iran, the findings of Exploratory Factor Analysis (EFA) based on Principal Component Analysis (PCA) technique also confirmed the factor structure of the fatigue scale. Results of discriminant analysis showed that FSS could distinguish fatigue between two groups of healthy subjects and MS patients from each other. Its overall classification accuracy is equal to 74.1%. Also, the mean of scores of all components related with quality of life and psychopathological symptoms in fatigued patients compared to non-fatigued ones was significantly greater considering the cutoff point of ≤4. ^21^ The ICC was reported 0.93 for the total score that showed high repeatability of FSS and the Cronbach’s alpha was reported 0.96. ^18^

The KDQOL-SF includes 43 items related to the quality of life in relation to renal patients, with 36 items related to general health. Specific dimensions of the questionnaire include: symptoms and the list of problems (12 items), the effect of kidney disease (8 items), the burden of kidney disease (4 items), job performance (2 items), cognitive function (3 items), the quality of social relationships (3 items), sexual function (2 items), sleep (4 items), social support (2 items), medical staff support (2 items), and general health status (1 item). ^22^ Different questions have different answer options. As to scoring, each question is scored in a scale ranging from 0 (worst health) to 100 (best health). ^23^ In the present study, the mean KDQOL-SF score for each individual was considered as the final index, which is derived from the sum of the individual scores from different domains and divided by the number of questions, with values ranging from 0 to 100.

Based on Senanayake et al.’s study (2017), convergent and divergent validity revealed satisfactory construct validity. The Cronbach’s alpha of all KDQOL-SF domains, with the exception of cognitive performance and social function, exceeded the Nunnally’s criteria of 0.7. The Intra-class Correlation Coefficients (ICC) was more than 0.8 for all domains, indicating good test-retest reliability. ^23^ In Tao et al.’s study (2014), the panel determined the content validity using a four-point Likert scale. The item-level content validity index (I-CVI) and scale-level CVI (S-CVI) were 1.0. With regard to convergent validity, significant positive correlations were found between all of the subscale scores and the overall health rating score (P<0.01). ^24^

Also, in the study of Yekaninezhad et al. (2012) in Iran, all of the dimensions in the questionnaire showed good test–retest reliability (all above 0.7). All of the dimensions met the minimal criteria (i.e. 0.7) for internal consistency, and Cronbach’s alpha ranged from 0.73 to 0.93. The questionnaire’s content validity was assessed by a panel consisting of specialists in nephrology, public health, nursing and 6 patients. For construct validity, Pearson correlation coefficient showed a significant relationship between all general and specific dimensions of SF-KDQOL questionnaire and general health rating scale (P<0.01). EFA by principal-component analysis with rotation of factors showed that the SF-KDQOL specific questionnaire was summarized in two factors. These two factors were named “burden of disease” and “patient satisfaction”. It is worth noting that these two factors described more than 63% of the variance of data. ^22^

Participants were randomly allocated into four groups to reduce the confounders. Both participants and researcher were blind to participant allocation; however, due to noticeable differences in the oils used in foot massage, it was not possible to blind the researcher who performed the foot massage intervention and participants. 

At all stages of the study, foot massage was performed only by the trained researcher, who learned foot massage techniques from a traditional medicine practitioner and received a certificate of foot massage at a recognized Iranian Traditional Medicine Association (ITMS). 

In the first group, foot massage was done with chamomile oil (*Anthemis Pseudocotula* in a Sesame Oil base, produced by Company Naushad). During hemodialysis, the researcher performed a foot massage on the participant one hour after the start of the hemodialysis, after washing and drying the participant’s legs in a supine position. The foot massage was performed on the thenar and thumb by briefly pressing as rotationally, from the heel to the toes, with 3 cc of chamomile oil for 20 minutes (10 minutes per foot); then, the legs were re-dried with paper towels. In this group, foot massage was performed on the soles of the feet three times a week (when they referred to dialysis) for a period of two months.

In the second group, the massage of the foot was performed with almond oil (*Prunus amygdalus bitter*, produced by Company Naushad). The foot massage followed the same protocol as the chamomile group, but we used 3 cc of almond oil instead. In the third group, dry massage of the feet without using any oil was performed, using the same protocol as groups 1 and 2. In the control group, there was no intervention and the participants were only monitored. Before and two months after completion of intervention, FSS and KDQOL-SF questionnaires were completed by the participants in the intervention and control groups. The questionnaires were distributed by the second researcher among the patients and completed by patients during their referral to hemodialysis ward of Taleghani and Imam Khomeini hospitals. All groups received regular hemodialysis care as indicated by their physicians. 

Data analysis was performed using SPSS V 16 (IBM Corp.) at the significant level of <0.05. We used ANOVA to test the equality of four group means, and Tukey’s post hoc test to find out which particular differences between the pairs of means were significant. Paired t-tests were used for comparison of pre- and post- data. The trial was approved by the Ethics Committee of Urmia University of Medical Sciences with the code number of Ir.umsu.rec.1395.181. The participants were informed and written consent was obtained before their participation in the study. Data confidentiality and anonymity was guaranteed for volunteers participating in the study. 

## RESULTS

The mean age of the patients participating in this study was 55.2±12.7 years and the mean duration of the disease was 4.70±2.53. There were no significant differences between the groups for any socio-demographic characteristics (P>0.05). Distribution of other socio-demographic characteristics in groups is shown in Table 1.

**Table 1:** Socio-demographic characteristics of the participants by the study groups

Characteristics	Chamomile oil group	Almond oil group	Without oil group	Control group	P value*
	N (%)	N (%)	N (%)	N (%)	
Marital status					
Married	27 (90)	27 (90)	25 (83.30)	24 (80)	0.60
Unmarried	3 (10)	3 (10)	5 (16.70)	6 (20)	
Education level (years)					
Under High school	11 (36.70)	11 (36.70)	13 (43.30)	10 (33.30)	0.87
High school or above	19 (63.30)	19 (63.30)	17 (56.70)	20 (66.70)	
Income level					
Less than enough	4 (13.30)	7 (23.30)	7 (23.30)	7 (23.30)	0.71
Enough and more	26 (86.70)	23 (76.70)	23 (76.70)	23 (76.70)	

* Chi-square

Before the intervention, mean baseline FSS scores were not significantly different across the four groups (P=0.3). However, the mean FSS scores after the implementation of the foot massage using either chamomile oil, almond oil and no oils in all intervention groups were significantly lower than the control group, and each intervention group had significantly greater reductions in FSS than the control group (P≤0.001). Comparisons of FSS by the study groups are shown in Table 2. There were no significant differences in FSS within the control group between the start and end of the study period (P=0.9). However, there were significant differences between pre- and post-intervention FSS scores in all intervention groups (P<0.001). According to Tukey’s test, the differences between the post-test fatigue mean score of the almond oil foot massage group and chamomile, without oil and control groups were statistically significant (P=0.04, P=0.01, P<0.001, respectively) (Table 3).

**Table 2:** Comparison of fatigue severity scores by the study groups

Fatigue severity	Chamomile oil group	Almond oil group	Without oil group	Control group	P value*
	Mean±SD	Mean±SD	Mean±SD	Mean±SD	
Pre-intervention	5.25±1.35	6.06±0.76	5.82±0.86	5.53±1.24	0.3
Post-intervention	4.52±1.34	4.57±1.25	5.21±1.22	5.51±1.17	0.005
Difference Post/Pre	-0.72±1.22	-1.48±1.23	-0.6±0.99	-0.02±1.22	0.001
P value**	0.001	0.001	0.001	0.9	

* ANOVA;

** Paired t-test

**Table 3:** Comparison of fatigue severity scores among the groups

Outcome	Chamomile oil vs. almond oil group	Chamomile oil vs. without oil group	Chamomile oil vs. control group	Almond oil vs. without oil group	Almond oil vs. control group	Without oil vs. control group
Mean difference	0.75	-0.12	-0.7	-0.88	-1.46	-0.58
P value*	0.04	0.97	0.07	0.01	0.001	0.18

* Tukey’s post hoc test

Before the intervention, there were no significant differences between mean KDQOL-SF scores between all four groups (P=0.8). Mean KDQOL-SF scores after the intervention in all intervention groups increased compared to the control group, but this increase was not statistically significant (P=0.34). One-way ANOVA between groups showed a statistically significant difference between the mean difference post-pre-intervention on KDQOL-SF scores (P<0.001). Paired t-test also showed that the differences between the pre-test and post-test KDQOL-SF mean score of all intervention groups were statistically significant (P<0.001). However, in the control group the difference was not statistically significant (P=0.06) (Table 4). According to Tukey’s test, the differences in the post-test KDQOL-SF mean score of the chamomile oil foot massage group and almond, without oil and control groups were statistically significant (P<0.001) (Table 5).

**Table 4:** Comparison of the quality of life scores by the study groups

Quality of life	Chamomile oil group	Almond oil group	Without oil group	Control group	P value*
	mean±SD	mean±SD	mean±SD	mean±SD	
Pre-intervention	49.50±11.80	51.10±16.10	47.60±11.20	48.90±14.40	0.8
Post-intervention	53.70±10.30	53.50±15.10	49±10.50	48.80±14.07	0.34
Difference Post / Pre	4.20±2.56	2.340±1.90	1.40±1.20	0.11±1.10	0.001
P value**	0.001	0.001	0.001	0.06	

* ANOVA;

** Paired t-test

**Table 5:** Comparison of the quality of life scores among the groups

Outcome	Chamomile oil vs. almond oil group	Chamomile oil vs. without oil group	Chamomile oil vs. control group	Almond oil vs. without oil group	Almond oil vs. control group	Without oil” group vs. control group
Mean difference	1.88	2.81	3.30	0.93	1.42	0.48
P value*	0.001	0.001	0.001	0.19	0.01	0.7

* Tukey’s post hoc test

## DISCUSSION

The present study revealed that foot massage with chamomile oil and almond oil applied to the hemodialysis patients in both control and massage groups for eight weeks and 24 sessions decreased fatigue. However, fatigue complaints in men who received massage were found to demonstrate a larger decrease when compared to the control group. Before the intervention, mean baseline FSS scores in each of the four groups indicated that the patients had high levels of fatigue. Fatigue occurs in hemodialysis patients who have CRF due to various causes such as accumulation of waste from metabolism in the body, abnormal energy consumption, anemia or numbness25 therefore, the majority of hemodialysis patients experience fatigue. ^26^ The fact that before the intervention, mean baseline FSS scores were not significantly different across the four groups shows that the groups’ fatigue levels were homogeneous. However, the mean FSS scores after the implementation of massage in all intervention groups were significantly lower than the control group, suggesting that foot massage reduced their fatigue.

The results of this study are consistent with those of other studies in the literature that have examined the effect of other complementary therapies on fatigue among hemodialysis patients. Reflexology, ^27^ foot reflexology and back massage ^28^ and aromatherapy ^26^ have shown improvement on fatigue in patients undergoing chronic hemodialysis. Foot reflexology and back massage improved the sleep quality and reduced fatigue in hemodialysis patients. Compared to back massage, foot reflexology was determined to be more effective. ^28^ In our study, foot massage with almond oil appeared to be the most effective foot massage treatment for fatigue in hemodialysis patients. This may be due to the softening, strengthening and watering properties of this material, which can affect the elasticity of skin collagen fibers. In a study which aimed to compare the effects of aroma hand massage with only hand massage on fatigue and sleep, the findings showed even though there was no significant difference in the effects of aroma hand massage with only hand massage on fatigue, the increase in the fatigue level in the aroma hand massage group was lower than the only hand massage group. ^29^ The findings of the mentioned study are inconsistent with those of the present study. However, it is necessary to note that this study differed in terms of methodology. 

In this study, the quality of life mean scores after the intervention increased in all intervention groups compared to the control group, but this difference was not statistically significant. We studied the research related to foot massage in the literature. In studies conducted with different patient groups, foot massage showed to reduce anxiety and blood pressure and increase the quality of life. ^30 , 31^ However, in comparison with these results, there are studies that show that foot massage does not have a significant effect. ^32^ This is according to a recent systematic review that reflects a lack of evidence regarding the effect of different non-pharmacological interventions on the quality of life and a difference in the results of various studies. ^33^ In the randomized controlled studies, the quality of life was not corrected with a four week massage therapy. ^34^ In another study, it was found that foot massage had no positive effect on agitation and stress reduction in patients with dementia. ^32^ The finding that – whilst all massage techniques appeared effective, the inclusion of different therapeutic oils resulted in different improvement is a finding that warrants further examination. According to our study, foot massage with chamomile oil appeared to be the most effective foot massage intervention. This may be due to the pharmacological properties of chamomile oil itself. ^13^ The impact that inclusion of botanical medicines has on the effectiveness of various massage techniques is a topic that warrants further examination. 

One of the strengths of the present study was its new approach to a treatment method, which involved no complications for controlling fatigue and improving quality of life in hemodialysis patients. The pre- and post- nature of this study also means that the results cannot be automatically implied to infer longer term benefit or cast light on the duration of benefit of the treatments. Nonetheless, as one of the first investigations into massage as an adjunct treatment in hemodialysis patients, this study offers a foundation far larger, more robust trial designs that provide this granularity of data. The findings of this study support the idea that foot massage is used as a nursing care method to reduce the patient’s hemodialysis problems and increase their quality of life, but there were several limitations to our study, and interpretation of the results should be viewed in this context. One of the potential limitations of our study is that the patients’ psychological conditions during the completion of the questionnaire might have affected the responses to the questionnaire; yet, these were not examined in detail. However, this problem was equal across all intervention and control groups, and the study was conducted in normal psychological conditions, and as such this problem may be reduced. 

## CONCLUSION

Foot massage appears to be effective in reducing fatigue severity and improving quality of life in hemodialysis patients. All forms of foot massage in this study were more effective in the test groups than the controls, but there were significant differences in foot massage with almond oil which was the most effective intervention at reducing fatigue and foot massage with chamomile oil being most effective at improving the quality of life, suggesting that their integration may be both feasible and practical. 

Furthermore, involvement of patients’ companions as family caregivers in massage therapy can lead to continuation of this effective intervention after discharge from the hospital. Of course, appropriate education and training must be considered by nurses with respect to patient’s safety and provision of effective care. Given the complications and heavy cost of managing complications in patients undergoing hemodialysis, it appears that these methods can be used as an inexpensive and effective treatment to reduce the complications in hemodialysis patients; further studies are recommended to assure the safety and effectiveness of the procedures.
